# Results of the non-small cell lung cancer part of a phase III, open-label, randomized trial evaluating topical corticosteroid therapy for facial acneiform dermatitis induced by EGFR inhibitors: stepwise rank down from potent corticosteroid (FAEISS study, NCCH-1512)

**DOI:** 10.1007/s00520-020-05765-7

**Published:** 2020-09-11

**Authors:** Kazumi Nishino, Yutaka Fujiwara, Yuichiro Ohe, Ryota Saito, Eisaku Miyauchi, Tetsu Kobayashi, Yasuo Nakai, Toshiaki Takahashi, Taro Shibata, Tetsuya Hamaguchi, Katsuko Kikuchi, Naoya Yamazaki, Haruhiko Fukuda, Keiko Nozawa, Yoshio Kiyohara

**Affiliations:** 1 Department of Thoracic Oncology, Osaka International Cancer Institute, Osaka, Japan; 2 Department of Thoracic Oncology, National Cancer Center Hospital, Tokyo, Japan; 3 Department of Respiratory Medicine, Mitsui Memorial Hospital, Tokyo, Japan; 4 Department of Respiratory Medicine, Tohoku University Hospital, Miyagi, Japan; 5 Department of Pulmonary and Critical Care Medicine, Mie University Graduate School of Medicine, Mie, Japan; 6 Department of Dermatology, Mie University Graduate School of Medicine, Mie, Japan; 7 Division of Thoracic Oncology, Shizuoka Cancer Center, Shizuoka, Japan; 8 Biostatistics Division, Center for Research Administration and Support, National Cancer Center, Tokyo, Japan; 9 Department of Gastroenterological Oncology, Saitama Medical University International Medical Center, Saitama, Japan; 10 Department of Dermatology, Tohoku University Graduate School of Medicine, Miyagi, Japan; 11 Sendai Taihaku Dermatology Clinic, Miyagi, Japan; 12 Department of Dermatological Oncology, National Cancer Center Hospital, Tokyo, Japan; 13 Data Management Division, National Cancer Center Hospital, Tokyo, Japan; 14 Appearance Support Center, National Cancer Center Hospital, Tokyo, Japan; 15 Department of Dermatology, Shizuoka Cancer Center, Shizuoka, Japan

**Keywords:** Facial acneiform rash, Non-small cell lung cancer, Epidermal growth factor receptor, Topical corticosteroid, Minocycline, Heparinoid moisturizer

## Abstract

**Purpose:**

This FAEISS study was designed to confirm the superior efficacy of reactive topical corticosteroid strategies employing serially ranking-DOWN from very strong steroid levels for the treatment of facial acneiform rash induced by epidermal growth factor receptor (EGFR) inhibitors (EGFRIs), in comparison with strategies employing serially ranking-UP from weak steroid levels. This article reports the primary results of the non-small cell lung cancer (NSCLC) part of the trial.

**Methods:**

Patients with *EGFR*-mutated advanced NSCLC treated with erlotinib or afatinib were enrolled in the first registration. All patients received preemptive therapy with oral minocycline and heparinoid moisturizer from the initiation of an EGFR inhibitor. Enrolled patients who developed facial acneiform rash within 2 weeks were randomized at second registration to either a ranking-UP (WEAK) group or a ranking-DOWN group. The primary endpoint was incidence of grade ≥ 2 facial acneiform rash over 8 weeks.

**Results:**

Fifty-one patients were enrolled at the first registration and received EGFRIs (*n* = 30 for afatinib, *n* = 21 for erlotinib). However, 35 patients did not develop facial acneiform rash within 2 weeks; one patient discontinued preemptive treatment. Fifteen patients (29.4%) were enrolled in the second registration; nine were assigned to the WEAK group and six to the DOWN group. There was no significant difference in the incidence of grade ≥ 2 facial acneiform rash between the WEAK group (one patient, twice) and the DOWN group (one patient, twice; *p* = 0.8417). No patients developed severe facial acneiform rash within 10 weeks.

**Conclusion:**

In NSCLC patients who received EGFRIs, preemptive therapy of oral minocycline and heparinoid moisturizer reduced facial acneiform rash incidence.

**Trial registration:**

UMIN000024113

## Introduction

Epidermal growth factor receptor (EGFR) inhibition is an established and effective treatment option for patients with non-small cell lung cancer (NSCLC) [1–4], colorectal cancer (CRC) [5, 6], and squamous cell carcinoma of the head and neck (SCCHN) [7]. Many EGFR inhibitors are approved for clinical use, including monoclonal antibodies (such as cetuximab, necitumumab, and panitumumab) and tyrosine kinase inhibitors (TKIs) (such as gefitinib, erlotinib, afatinib, dacomitinib, and osimertinib). Although EGFR inhibitors improve patient survival, they are also associated with skin toxicity, including acneiform rash, xerosis, paronychia, and pruritus, and hair, eyelash, and mucosal changes [8–10].

The incidence of facial acneiform rash of all grades has been estimated to be 60–90% from previous clinical trials [11, 12]. Although these adverse reactions are rarely serious or life-threatening, skin toxicity can significantly affect quality of life (QOL), causing both physical discomfort and psychological distress, which can affect compliance, thus affecting the efficacy of treatment with EGFR inhibitors [13–16]. Therefore, proper management of these toxicities is essential. Lacouture et al. reported that preemptive treatment, including skin moisturizers, sunscreen, topical steroid, and doxycycline, could reduce the severity of EGFR inhibitor-associated skin toxicities in patients with metastatic CRC [17]. Since the application site for these topical steroids is mainly on the face, there is concern over the side effects of steroids due to long-term use [18]. Therefore, few physicians currently recommend long-term, prophylactic topical steroid treatment on the face. Since EGFR-TKIs induce less skin toxicity than EGFR antibodies, patients with NSCLC harboring *EGFR* mutations are generally treated only with preventative moisturizers, and with reactive oral minocycline and topical steroids only at the onset of the rash.

This FAEISS study (NCCH1512) was designed to confirm the superior efficacy of reactive topical corticosteroid strategies employing serially ranking-DOWN from very strong topical steroids (compared with strategies employing serially ranking-UP from weak topical steroids) for facial acneiform rash induced by EGFR inhibitors in metastatic NSCLC and CRC. Here, we report the primary results of the NSCLC part of a randomized controlled trial evaluating reactive topical corticosteroid strategies in patients with NSCLC and CRC.

## Patients and methods

### Participants

FAEISS was a randomized, open-label, multicenter, phase III study implemented in ten centers across Japan. Eligible patients were *EGFR*-mutant NSCLC patients who were to be treated with erlotinib or afatinib, and wild-type *KRAS* and *NRAS* patients with metastatic CRC who were to be treated with cetuximab or panitumumab. This paper describes the results obtained for patients with NSCLC. The other key eligibility criteria were age 20 to 79 years; Eastern Cooperative Oncology Group performance status (PS) 0 to 1; and adequate organ function.

### Study design and treatment schedule

The anticancer treatment was initiated with an investigator-choice EGFR-TKI (erlotinib or afatinib) at the recommended dose. All patients received 2 weeks of prophylactic treatment with minocycline (orally, 100 or 200 mg, per day) and heparinoid moisturizer at initiation of EGFR-TKI therapy. The dose of minocycline was an investigator’s choice. If facial acneiform rash was observed during the first 2 weeks, treatment of the rash with weak class topical corticosteroids was initiated. Patients who developed facial acneiform rash during the first 2 weeks were enrolled into the second registration.

**Fig. 1:**
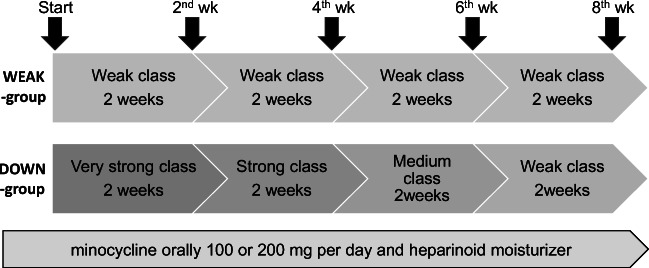
Study design for the multicenter, open-label, randomized, phase III trial for patients receiving EGFR inhibitors. All patients received preemptive therapy with oral minocycline (100 or 200 mg/day) and heparinoid moisturizer from the initiation of treatment with EGFR inhibitors. Enrolled patients who developed facial acneiform rash within 2 weeks were randomized either to ranking-UP group (WEAK group) or ranking-DOWN group (DOWN group) following second registration

Patients enrolled into the second registration were randomized to either the WEAK group (ranking-up) or the DOWN group (ranking-down) of reactive topical corticosteroid. The randomization procedure was accomplished using a minimization method balancing institution, type of EGFR inhibitor, and sex, in addition to adjustment factors. The “control treatment” (WEAK group) was defined as a reactive treatment that started with weak topical corticosteroids. In the control treatment group, if symptoms became worse, the reactive treatment was switched to stronger class corticosteroids. On the other hand, in the DOWN group, after the onset of facial acneiform rash, twice daily reactive treatment with very strong topical corticosteroids was started, and the strength of the steroid was gradually decreased every 2 weeks (to strong, then medium, then weak strengths). The types of topical steroids were as follows for each class. Prednisolone is a weak class and hydrocortisone butyrate is a medium class. A strong class is betamethasone valerate/gentamicin sulfate, or dexamethasone propionate. A “very strong” class is diflucortolone valerate, difluprednate, or betamethasone butyrate propionate. All patients continued prophylactic treatment with oral minocycline and heparinoid moisturizer after the second registration (Fig. 1). The evaluations for facial acneiform rash were performed five times beginning from 2 weeks after the first registration (and finishing at 10 weeks). Treatment after closing accrual of NSCLC patients was performed at the discretion of the investigators.

Photographic images of each patient’s face were taken at the participating site. All photos were collected centrally and subsequently reviewed by three dermatologists blinded to treatment status (central review). The reviewers assessed the severity of facial acneiform rash using an adapted grading system developed by Socpe et al. [19]. The adaptive grading system comprised grade 0–grade 3, representing none, mild, moderate, and severe rash.

### Outcomes

The primary endpoint was a direct comparison of the incidence of grade 2 (moderate) or higher facial acneiform rash in the WEAK and DOWN groups during the 10-week skin treatment period (by central review). Key secondary endpoints included the proportion of grade 2 or higher facial acneiform rash, the proportion of grade 3 (severe) or higher facial acneiform rash, the proportion of switching EGFR therapies due to facial acneiform rash, the proportion of continuing EGFR therapy at the end of protocol treatment, the proportion of grade 1 (mild) or higher facial acneiform rash at the end of protocol treatment, the incidence of adverse events on the face, and the proportion of stable QOL scores.

### Statistical considerations

The main clinical hypothesis of this study was that reactive treatment of facial acneiform rash during administration of EGFR inhibitors is more effective when starting with very strong topical corticosteroids than when starting with weak topical corticosteroids. The primary endpoint, the incidence of grade 2 or higher facial acneiform rash in each group, is assumed to be significantly lower in the DOWN group than in the WEAK group. Moreover, a key secondary endpoint, the incidence of adverse events on the face, is clearly assumed not to exceed in the WEAK group compared with that in the DOWN group. If these two endpoints are met, we may conclude that reactive treatment starting with very strong topical corticosteroids is more effective against facial acneiform rash.

A superiority trial design was applied to detect whether the incidence of grade 2 or higher facial acneiform rash in the DOWN group was less than that in the WEAK group. To provide the Wilcoxon rank sum test with 80% power at a 5% significance level (one-sided), 94 subjects over both groups (47 subjects per group) were required. Assuming facial acneiform rash (grade 1 or higher) occurs in 85% of all enrolled patients at the first registration, and that all affected patients progress to the second registration, 118 patients were required for the first registration. The planned sample size was set to be 120 patients. However, NSCLC patient accrual was closed in the middle of the accrual period since most NSCLC patients enrolled at the first registration did not develop facial acneiform rash within 2 weeks after initiation of EGFR-TKI treatment (and thus did not enroll in the second registration). Due to the non-confirmatory nature of the analysis for the NSCLC part of the trial, a two-sided *p* value is provided for the primary endpoint in this study. This trial is registered in UMIN-CTR (www.umin.ac.jp/ctr/) with identification number UMIN000024113.

## Results

**Fig. 2:**
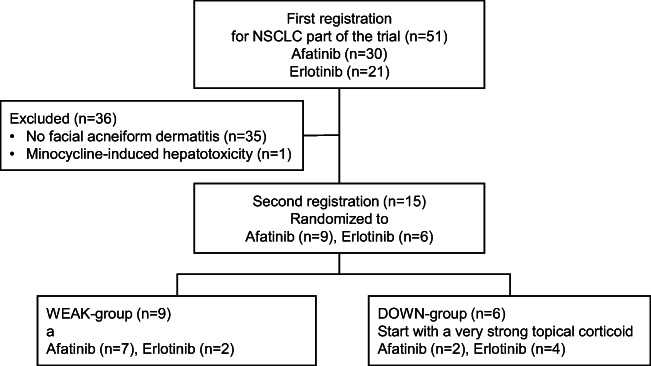
CONSORT diagram

From November 2016 to August 2018, 51 patients with NSCLC harboring *EGFR* mutations were enrolled for the NSCLC part of the trial in the first registration. All 51 patients received afatinib (*n* = 30) or erlotinib (*n* = 21) treatment. Thirty-four patients were female and 17 were male, and the median age was 66 years (range: 39–79). Thirty-five patients (68.6%) did not develop a facial acneiform rash within 2 weeks, while one patient discontinued preemptive treatment with minocycline due to hepatotoxicity. Therefore, only 15 patients (27.4%) were enrolled in the second registration; nine patients were assigned to the WEAK group and six patients to the DOWN group (Fig. 2). The baseline characteristics of these patients are listed in Table 1. NSCLC patient accrual was subsequently closed because patients proceeding to the second registration were fewer than assumed. Thus, the planned number of patients required for the primary analysis could not be obtained.

**Table 1:** Baseline characteristics

	First registration (*n* = 51)	Second registration (*n* = 15)	
		WEAK group	DOWN group
		(*n* = 9)	(*n* = 6)
Age			
Median (range) (year)	67 (39–79)	58 (45–78)	65 (52–76)
< 65 years—no. (%)	20 (39.2)	6 (66.7)	3 (50.0)
≥ 65 years—no. (%)	31 (60.8)	3 (33.3)	3 (50.0)
Sex—no. (%)			
Male	17 (33.3)	3 (33.3)	3 (50.0)
Female	34 (66.7)	6 (66.7)	3 (50.0)
ECOG performance status—no. (%)			
0	34 (66.7)	8 (88.9)	5 (83.3)
1	17 (33.3)	1 (11.1)	1 (16.7)
Stage			
IIIB	1 (2.0)	0	0
IV	38 (74.5)	6 (66.7)	4 (66.7)
Recurrence	12 (23.5)	3 (33.3)	2 (33.3)
Type of EGFR-TKIs—no. (%)			
Erlotiinib	21 (41.2)	2 (25.0)	4 (66.7)
Afatinib	30 (58.8)	7 (75.0)	2 (33.3)
Minocycline dosage—no. (%)			
100 mg/day	39 (76.5)	7 (77.8)	3 (50.0)
200 mg/day	10 (19.6)	1 (11.1)	3 (50.0)
None	2 (3.9)	1 (11.1)	0
Efficacy of an EGFR-TKI—no. (%)			
PR	20 (39.2)	6 (66.7)	2 (33.3)
SD	12 (23.5)	0	2 (33.3)
PD	5 (9.8)	1 (11.1)	1 (16.7)
NE	11 (21.6)	2 (22.2)	1 (16.7)
Unknown	3 (5.9)	0	0

**Fig. 3:**
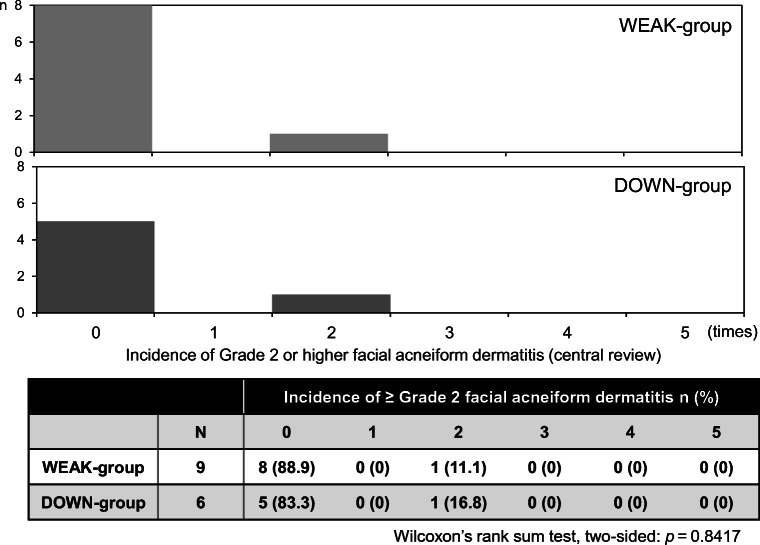
Incidence of grade 2 or higher facial acneiform dermatitis (central review)

There was no significant difference in the incidence of grade ≥ 2 facial acneiform rash between the WEAK group (one patient, twice) and the DOWN group (one patient, twice) (Wilcoxon’s rank sum test, two-sided: *p* = 0.8417) (Fig. 3). The proportion of grade ≥ 2 facial acneiform rash observed in the WEAK and DOWN groups were 11.1% (1/9) and 16.7% (1/6), respectively. No patients developed severe (grade ≥ 3) facial acneiform rash. Therefore, no patient in either group switched EGFR therapies due to facial acneiform rash. The proportion of patients continuing EGFR therapy at the end of protocol treatment was 66.7% (6/9) in the WEAK group and 50% (3/6) in the DOWN group. At the end of protocol treatment, the proportion of mild (grade ≥ 1) facial acneiform rash was 55.6% (5/9) in the WEAK group and 50% (3/6) in the DOWN group. Most patients immediately improved with topical corticosteroids, regardless of steroid strength.

Skin adverse events after the second registration (*n* = 15) included non-facial acneiform rash (*n* = 14); pruritus (*n* = 8); paronychia (*n* = 5); and dry skin (*n* = 3). Major non-skin adverse events included diarrhea (grade 3, *n* = 2); an increase in alanine aminotransferase (grade 2, *n* = 2), stomatitis (grade 3, *n* = 1), and pneumonitis (grade 1, *n* = 1).

## Discussion

In the present study, we were unable to confirm the superior efficacy of topical corticosteroids that started at very strong levels for facial acneiform rash when NSCLC patients were treated with EGFR-TKIs. This was because preemptive therapy with oral minocycline and heparinoid moisturizers resulted in an unexpectedly low rate of facial acneiform rash (expected 85% vs. actual 27.4%). However, even though it is not a prespecified hypothesis and this study was not powered for it, low incidence of developing facial acneiform rash is considered to be an evidence that preemptive therapy with oral minocycline and heparinoid moisturizer in NSCLC patients receiving EGFR-TKIs can reduce the incidence and severity of facial acneiform rash. After initiating prophylactic treatment at the start of treatment with EGFR-TKIs, the incidence of facial acneiform rash was decreased, and any subsequent appearance of facial acneiform rash could immediately be treated with reactive topical corticosteroids (regardless of the strength of the steroid).

Presently, five EGFR-TKIs (gefitinib, erlotinib, afatinib, dacomitinib, and osimertinib) are approved by the US Food and Drug Administration (FDA) and the Japanese Pharmaceuticals and Medical Devices Agency (PMDA) for the first-line treatment of patients with metastatic NSCLC harboring *EGFR* mutations. The safety and tolerability profiles of first- and second-generation EGFR-TKIs (gefitinib, erlotinib, afatinib, and dacomitinib) are well characterized. Treatment with these inhibitors is associated with skin toxicities such as acneiform rash, pruritus, xerosis, and paronychia. The dermatologic adverse events associated with osimertinib appear to be milder than those of first- and second-generation agents [20, 21]. The incidence of skin rash was 66–71% with gefitinib, 73–99% with erlotinib, 81–100% with afatinib, and 34–58% with osimertinib [11, 21]. In the present study, erlotinib and afatinib treatment regimens (which show higher skin toxicity) were investigated in order to better analyze the effects of topical steroids on the treatment of EGFR-TKI-induced acneiform rash.

Takeda et al. previously reported a pooled analysis of the incidence of acneiform rash (grade ≥ 3) during treatment with EGFR-TKIs (afatinib, 15% vs. erlotinib, 8.8%) [22]. According to the same study, the most common adverse events leading to withdrawal from EGFR-TKI treatment were related to skin toxicity [22]. In the Japanese post marketing surveillance study of erlotinib-treated patients with NSCLC (POLARSTAR study, *n* = 3488), median time to onset of rash was 8 days (range 1–494) [23].

Previous data concerning the use of topical steroids to treat acneiform rash induced by EGFR inhibitors are limited, and no standard treatment has been established. Published protocols concerning the use of topical steroids can be roughly divided into *prophylactic treatments* (beginning at the start of EGFR inhibitor administration) and *reactive treatments* (beginning after the onset of rash). Randomized trials of prophylactic treatments and reactive treatments were conducted for patients with metastatic CRC in the USA (STEPP) [17] and Japan (J-STEPP) [24]. In the STEPP study, Lacouture et al. reported that preemptive skin treatment might reduce the incidence of grade 2 or higher skin toxicities (compared with reactive treatment). These preemptive treatments included the use of skin moisturizers, sunscreen, topical steroids (1% hydrocortisone cream: weak class), and doxycycline [17]. In the J-STEPP study, preemptive skin treatment could reduce the severity of skin toxicities during panitumumab treatment in an Asian population with mCRC [24].

However, since the start time of antibiotic treatment (doxycycline/minocycline) in these trials was also different between the reactive and preemptive treatment groups, it was unclear if prophylactic use of topical steroids was itself beneficial. Moreover, since the application site for topical steroids is mainly on the face, the skin on the face may become thin following long-term use, and steroid acne may be induced. This is potentially an issue during longer term prophylactic administration of topical steroids. Indeed, the use of prophylactic topical steroids is rarely recommended by physicians.

Therefore, in the present study, unlike in the STEPP and J-STEPP trials, prophylactic treatment was initiated by administering oral minocycline and moisturizer at the start of EGFR inhibitors. While preemptive therapy for CRC patients is a standard treatment in Japan, this is not usually the case for NSCLC patients. Although prophylactical sunscreen was used in the STEPP and J-STEPP studies, prophylactical sunscreen was not used in the present study. This was because Japanese individuals normally require less UV protection than individuals of lighter-skinned races.

Five classes of topical steroid are marketed in Japan based on their anti-inflammatory potency: weak (e.g., prednisolone, 0.5%); medium (e.g., hydrocortisone butyrate); strong (e.g., betamethasone valerate); very strong (e.g., diflucortolone valerate); and strongest (e.g., clobetasol propionate). Kiyohara et al. recommended that strong or very strong topical corticosteroids be used for Japanese patients in the event of acneiform rash; in severe cases (grade 2/3), the strongest class of topical corticosteroids should be applied twice daily. Once symptoms have improved, the patient should be switched to a medium-class steroid. If symptoms become worse, the patient should be switched to stronger class steroids [25].

To date, most physicians do not provide prophylactic treatment for *EGFR* mutation-positive NSCLC patients at the start of EGFR-TKI treatment. Due to concerns about side effects such as interstitial pneumonia and hepatotoxicity caused by minocycline, they do not use in combination with an EGFR-TKI. However, there was no increase in side effects from minocycline in this study and it is worth considering preventive treatment with minocycline and moisturizers in patients with NSCLC. Regarding the amount of minocycline, rash occurred in 10 of 39 patients (26%) at 100 mg and 4 of 10 patients (40%) at 200 mg, and they were enrolled in the second registration (Table 1). This suggests that at least 200 mg minocycline is not always more effective than 100 mg. It is hoped that future studies will be conducted to select patients at risk for treatment, rather than administering it to all patients. Melosky et al. report that prophylactic minocycline and reactive treatment are both acceptable options for the treatment of erlotinib-induced rash in the second- or third-line setting of metastatic NSCLC (Pan Canadian Rash Trial) [26].

In the present study, although 51 patients with NSCLC were enrolled in the first registration, only 15 (27.4%) were enrolled in the second registration. Moreover, most patients showed immediate improvement following reactive treatment with topical corticosteroids, regardless of steroid strength. No patients developed severe facial acneiform rash (grade 3 or higher) within 10 weeks. In conclusion, in NSCLC patients who received EGFR-TKIs, preemptive therapy of oral minocycline and heparinoid moisturizer were effective in preventing facial acneiform rash.

## Data Availability

All data generated or analyzed during this study are included in this published article.
